# Supplementary material for: The role of PNI to predict survival in advanced hepatocellular carcinoma treated with Sorafenib
Source: PLoS One. 2020 May 7;15(5):e0232449. doi: 10.1371/journal.pone.0232449 (PMC7205300; doi:10.1371/journal.pone.0232449)
Supplement: Attachment — Submitted filename: reviewers.docx [file pone.0232449.s002.docx]

**Editor comment:** In your revised manuscript you should clarly highlight and discuss the novelty and differential value of your present study in view of previous works from your laboratory, such as Gastrointest Tumors, 2019;6:71-80  [doi.org/10.1159/000501593](http://doi.org/10.1159/000501593)

**REPLY: This study was different respect others our works because we evaluated here a simpler index than the previous. For example, RAPID index was composed about neutrophil, lymphocyte count, LDH and AFP, different PNI was com posed only by lymphocyte count and albumin. This simplification of the index makes it more available and simpler for daily clinical practice. Furthermore, we have increase the number and cohorts of patients than the previous studies. We have added this points in the discussion.**

**Reviewer #1:** The Authors report the results of a retrospective study aimed to investigate the role of the prognostic nutritional index (PNI) on survival in patients with advanced hepatocellular carcinoma (HCC) treated with sorafenib. The topic is of interest considering the expanding landscape of treatment options for advanced HCC and the need to identify predictive and prognostic markers that can be of help in selecting patients to be treated with the available drugs. However, the retrospective nature of the study and the lack of a validated cutoff value for the PNI limit the robustness of the results.

The following comments should be addressed.

General comment
1.     The English language should be revised

**REPLY: We have improved the English language of the paper**

Major comments:
1.     Introduction, page 4: Among the prognostic factors the Authors should mention the pattern of progression and the reason for sorafenib discontinuation as published by Reig M et al and Iavarone M et al, respectively (Hepatology 2013; Hepatology 2015). These factors should be included in the analyses

**REPLY: WE have added in the introduction these two papers. We have performed the analysis about the reason for sorafenib discontinuation. Unfortunately, we didn’t have collected data about Pattern of progression for this reason the analyzes could not be performed. We have added this point in the limitation section.**

2.     Results, Prognostic and predictive value of the PNI in training cohort: Did the Authors evaluate the predictive role of PNI or only the prognostic role? In the methods they stated that both have been assessed but no results are available for the predictive role. This aspect should be clarified and discussed in the discussion

**REPLY: We very thank the reviewer for this point. In absence of control arm is not possible to definite the prognostic role for this reason we have delete the sentence in the methods. We have added this point in the discussion.**

3.     Results, Validation cohorts: Baseline characteristics are partly different (e.g., ECOG PS, etiology) or missing (e.g., ALBI grade) in the validation cohorts. Did the Authors consider this factor in their analysis? This point should be also discussed in the discussion among the limitations of the study

**REPLY: WE completely agree with the reviewer for this point. We have added it in the limitation section.**

4.     References: The number of references from the same Authors should be reduced and references from other groups should be added

**REPLY: We have reduced our references.**

Minor comment:
1.     References: Ref #1 should be replaced with a more recent paper

**REPLY: We have changed the reference 1.**

**Reviewer #2:** I read with interest the manuscript by Caputo et al. on the role of PNI to predict survival in advanced hepatocellular carcinoma treated with Sorafenib. Authors define the optimal cut-off of PNI independently associated with survival in a training cohort, and validate the finding in two external validation cohorts.

I have only the following minor points:
1) It was surprising to me seeing that, in the training cohort, neither ALBI grade nor Child-Pugh class nor BCLC class were associated with prognosis at the univariate analysis. It is probably due to relatively small number of patients in the worst classes. Do Authors have any other explication for this? In any case, I think this should be properly acknowledged and discussed.

**REPLY: we observed a trend towards a worse prognosis for these patients. We agree with the reviewer that it was a consequence of a small number of patients. We have added this point in the result.**

2) In the Abstract, Results, the median OS of patients with low and high PNI has been inverted.

**REPLY: We very thank the reviewer for this point.**

3) In The Methods, Authors state, among the eligibility criteria, there was a Child-Pugh liver class A. However, subsequently, it is clearly reported that also Child-Pugh B patients have been enrolled in the 3 cohorts.

**REPLY: Yes, we completely agree with the reviewer, we enrolled patients in real life. We have rewritten eligibility criteria of the study.**
